# Supplementary material for: Live imaging of delamination in Drosophila shows epithelial cell motility and invasiveness are independently regulated
Source: Sci Rep. 2022 Sep 28;12:16210. doi: 10.1038/s41598-022-20492-1 (PMC9519887; doi:10.1038/s41598-022-20492-1)
Supplement: Supplementary file 1 — Supplementary Information 1. [file 41598_2022_20492_MOESM1_ESM.pdf]

Supplementary Information

Live imaging of delamination in Drosophila shows epithelial cell motility and invasiveness are independently regulated

Mikiko Inaki, Smitha Vishnu and Kenji Matsuno

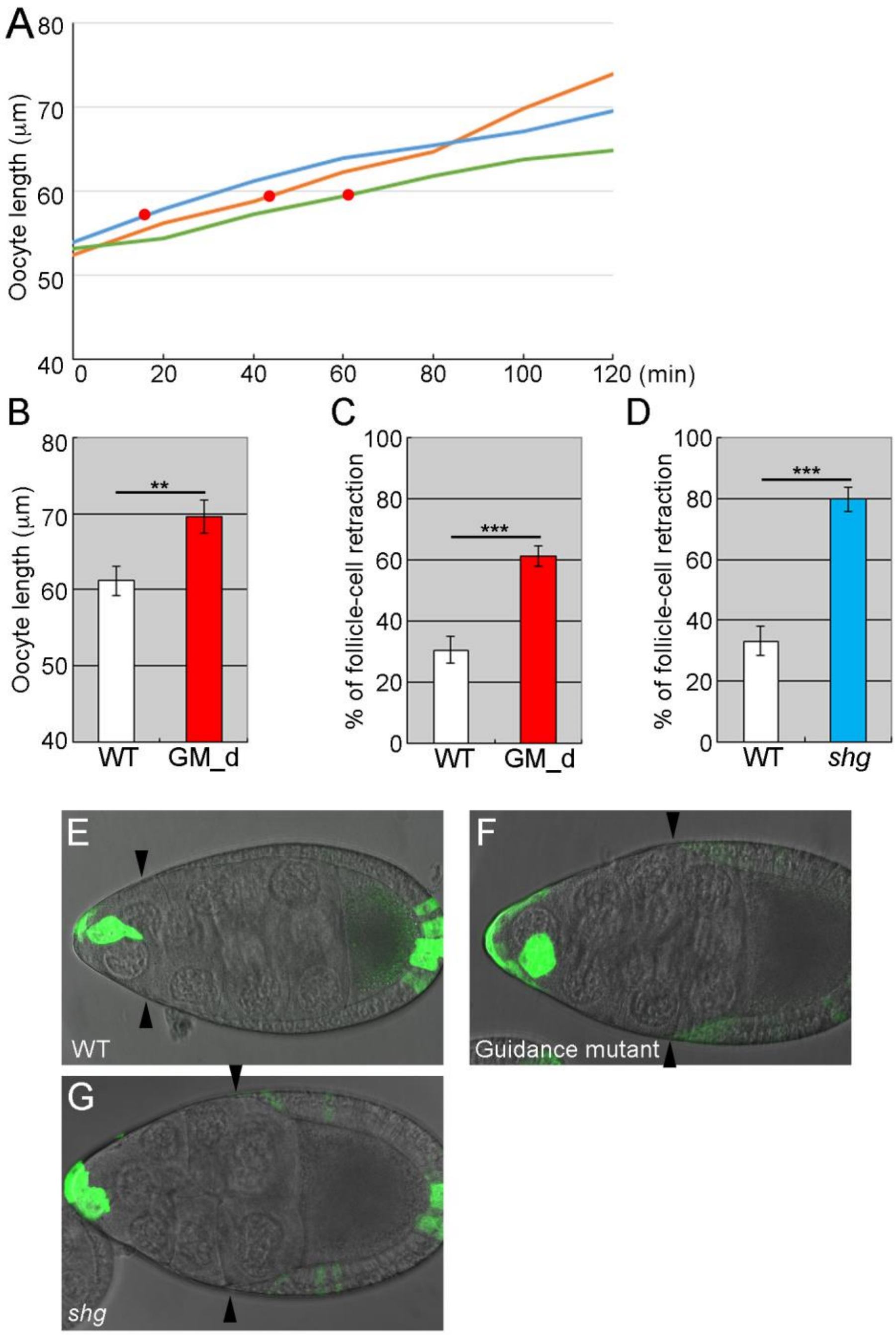

Fig. S1

(A) A graph of oocyte length measured over time. Each line shows oocyte length for a different wild-type embryo, measured from three representative movies. Elapsed time from the beginning

of the movie is shown at the bottom of the graph. Red circles indicate the delamination point for each embryo. (B, C) Bar graphs show oocyte length (B) and the % of follicle-cell retraction (C) at the delamination point in wild-type (WT) and guidance-mutant (GM_d) egg chambers. In B, oocyte length was measured in only 5 of 11 guidance-mutant embryos since the oocyte grew too long to stay in frame in the remaining 6 embryos and thus could not be measured. $5 \leqq N \leqq 18$. Error bars indicate SEM. (D) A bar graph showing the % of follicle-cell retraction at the delamination point in WT (*slbo*-gal4, UAS-CD8-GFP) and *shg* (shg^{PB4354}/ shg^{PB4354}; *slbo*-gal4, UAS-CD8-GFP) embryos. For *shg* mutants, oocyte length could not be measured at the delamination point because the oocytes had grown too long to stay in frame. $3 \leqq N \leqq 4$. Error bars indicate SEM. (E-G) Transmission and GFP (driven by *slbo*-gal4, green) images of wild-type (E) and guidance-mutant (F) egg chambers at the delamination point, and the delaminating *shg*-mutant egg chamber (G). Black arrowheads indicate the edge of retracting follicle cells. For all images, anterior is left.

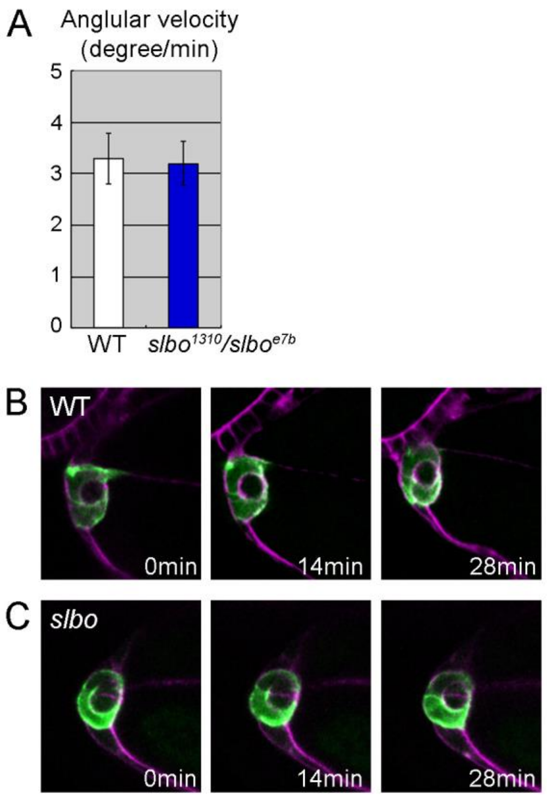

Fig. S2

(A) A bar graph showing the angular velocity of wild-type (WT) and *slbo*^{1310}/*slbo*^{e7b} border cells; angular velocities were comparable in these conditions. Number of cells (n) =10, number of egg chambers (N) =5. Error bars indicate SEM. (B,C) F-actin was visualized by Lifeact-GFP (green) in movies of wild-type (B) and *slbo*^{1310}/*slbo*^{e7b} (C) border cells showing locomotive behaviors. Elapsed time from the start of the movie is shown in the lower right corner. For all images, anterior is left.

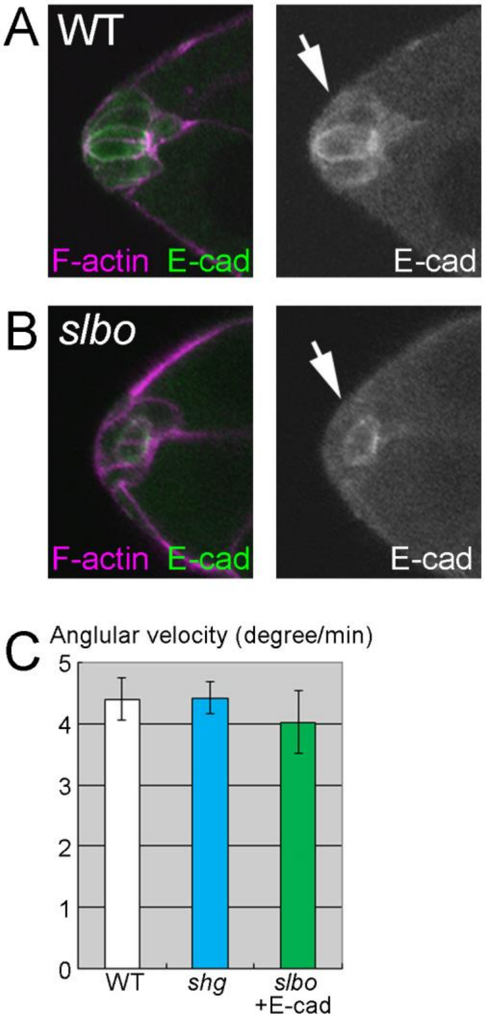

Fig. S3

(A,B) Distribution of E-cad (green) and F-actin visualized by Phalloidin staining (magenta) in delaminating wild-type (A) and *slbo*^{1310}/*slbo*^{1310} (B) border cells in fixed samples. White arrows indicate E-cad expression in border cells. E-cad expression was similar in *slbo*-mutant and wild-type polar and nurse cells but was lower in *slbo*-mutant border cells than in wild-type. For all images, anterior is left. (C) A bar graph showing the angular velocity of border cells in wild-type,

shg-mutant (*shg*), and *slbo*-mutant with E-cad (*slbo*+E-cad) embryos. Angular velocity was comparable in these conditions. $10 \leqq n \leqq 26$, $5 \leqq N \leqq 13$. Error bars indicate SEM.

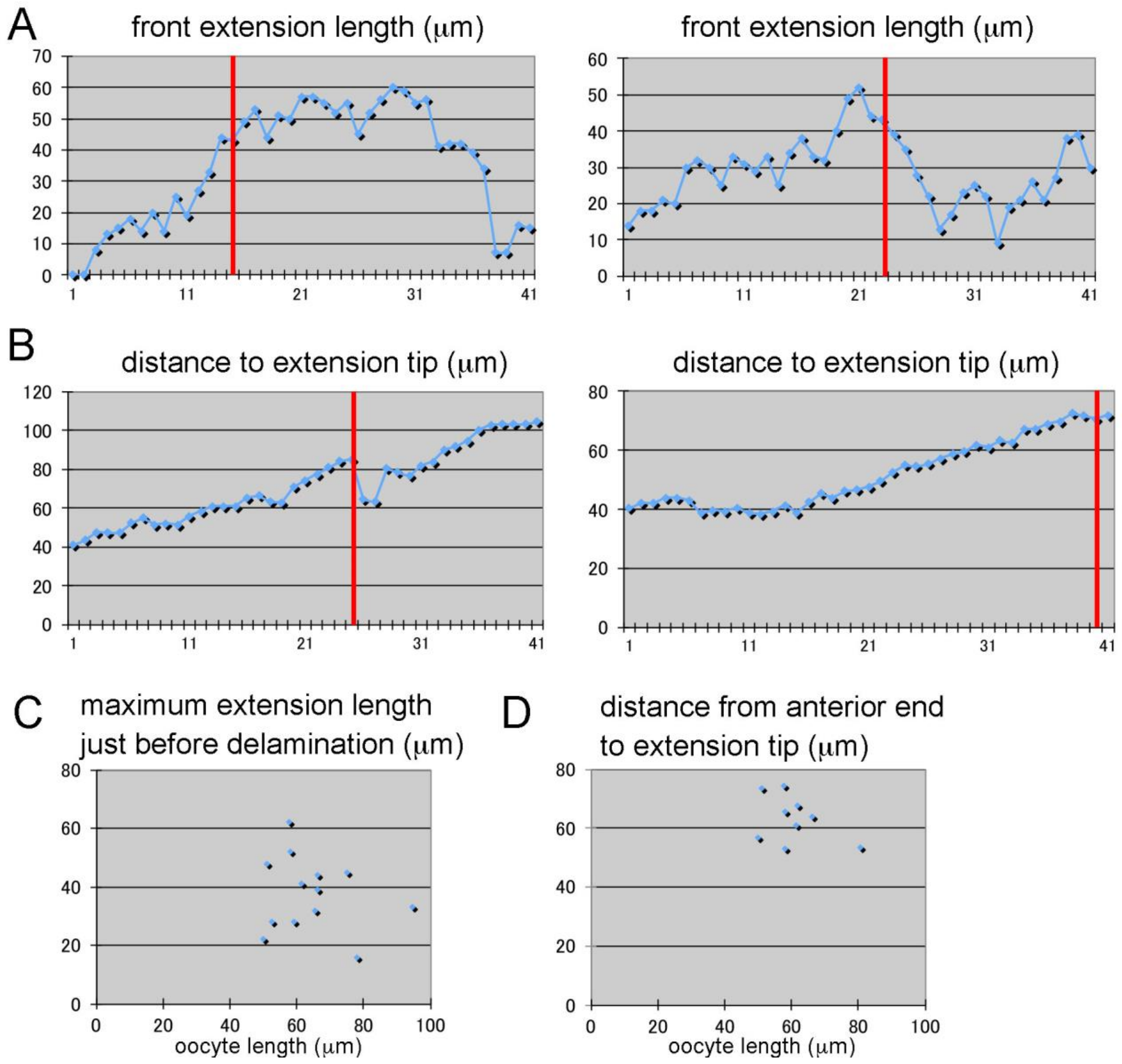

Fig. S4

(A,B) Examples of fluctuations in front extension length (A) and in cluster reach, defined as the distance between the anterior end of the egg chamber and the extension tip (B), during delamination. Time elapsed (min) from the the start of the movie is shown at the left. Red lines show the delamination point. Front extension length varied quite a bit, while cluster reach tended

to increase over time. The maximum extension length did not correlate with the delamination point. (C,D) The maximum extension length (C) and cluster reach (D) just before delamination. Length varied more than reach.

Movies

Movie 1

Delaminating wild-type *Drosophila* border cells form a cluster of jiggling cells. The cluster extends a protrusion in the direction of future migration, and the tip finds an attachment point. The cell cluster detaches from the epithelial layer and follows the protrusion as it contracts. Elapsed time (minutes) shows at the lower right.

Movie 2

JAK/STAT-inhibited follicle cells failed to differentiate into border cells, move into clusters, or extend protrusions in the period corresponding to wild-type delamination. JAK/STAT signaling was inhibited with DOME lacking a cytoplasmic domain. Elapsed time shows at the lower right.

Movie 3

slbo-mutant border cells formed clusters and moved within the clusters, but did not form protrusions or delaminate during the period when wild-type cells would delaminate. Elapsed time shows at the lower right.

Movie 4

slbo-mutant border cells formed clusters but lost motility when treated with CytochalasinD, which inhibits actin polymerization, and failed to delaminate during the wild-type delamination period. Elapsed time shows at the lower right.

Movie 5

shg-mutant border cells formed clusters of motile cells and sent out short extensions, but did not delaminate during the wild-type delamination period. Elapsed time shows at the lower right.

Movie 6

slbo-mutant border cells with E-cad expression formed front extensions but did not delaminate during the wild-type delamination period. Elapsed time shows at the lower right.

Movie 7

Although the movements of guidance-deficient and wild-type border cells were similar, front extensions were shorter and less persistent in guidance-deficient tthan in wild-type extensions.

Movie 8

Guidance-deficient border cells delaminated successfully. Elapsed time shows at the lower right.

Movie 9

E-cad expression rescued invasive extensions in JAK/STAT-inhibited border cells but did not rescue cell movement. Elapsed time shows at the lower right.

Movie 10

Slbo overexpression induced front extensions in JAK/STAT-inhibited follicle-like cells, but the cells failed to delaminate. Elapsed time shows at the lower right.
